# On the Mechanism of Synaptic Depression Induced by CaMKIIN, an Endogenous Inhibitor of CaMKII

**DOI:** 10.1371/journal.pone.0049293

**Published:** 2012-11-08

**Authors:** Camilo Gouet, Belen Aburto, Cecilia Vergara, Magdalena Sanhueza

**Affiliations:** 1 Department of Biology, Faculty of Sciences, University of Chile, Santiago, Chile; 2 Millennium Institute for Cell Dynamics and Biotechnology, University of Chile, Santiago, Chile; Univ. Kentucky, United States of America

## Abstract

Activity-dependent synaptic plasticity underlies, at least in part, learning and memory processes. NMDA receptor (NMDAR)-dependent long-term potentiation (LTP) is a major synaptic plasticity model. During LTP induction, Ca^2+^/calmodulin-dependent protein kinase II (CaMKII) is activated, autophosphorylated and persistently translocated to the postsynaptic density, where it binds to the NMDAR. If any of these steps is inhibited, LTP is disrupted. The endogenous CaMKII inhibitor proteins CaMKIINα,β are rapidly upregulated in specific brain regions after learning. We recently showed that transient application of peptides derived from CaMKIINα (CN peptides) persistently depresses synaptic strength and reverses LTP saturation, as it allows further LTP induction in previously saturated pathways. The treatment disrupts basal CaMKII-NMDAR interaction and decreases bound CaMKII fraction in spines. To unravel CaMKIIN function and to further understand CaMKII role in synaptic strength maintenance, here we more deeply investigated the mechanism of synaptic depression induced by CN peptides (CN-depression) in rat hippocampal slices. We showed that CN-depression does not require glutamatergic synaptic activity or Ca^2+^ signaling, thus discarding unspecific triggering of activity-dependent long-term depression (LTD) in slices. Moreover, occlusion experiments revealed that CN-depression and NMDAR-LTD have different expression mechanisms. We showed that CN-depression does not involve complex metabolic pathways including protein synthesis or proteasome-mediated degradation. Remarkably, CN-depression cannot be resolved in neonate rats, for which CaMKII is mostly cytosolic and virtually absent at the postsynaptic densities. Overall, our results support a direct effect of CN peptides on synaptic CaMKII-NMDAR binding and suggest that CaMKIINα,β could be critical plasticity-related proteins that may operate as cell-wide homeostatic regulators preventing saturation of LTP mechanisms or may selectively erase LTP-induced traces in specific groups of synapses.

## Introduction

The multifunctional holoenzyme CaMKII plays a critical role in NMDAR-dependent LTP and memory formation [1], [2]. LTP induction involves Ca^2+^ influx through NMDARs and activation of CaMKII, that translocates to stimulated spines and postsynaptic densities (PSD) [3]–[5], regulating AMPA-receptor (AMPAR) localization and function. A key binding partner of CaMKII at PSD is the NMDAR subunit NR2B [6], [7]. CaMKII undergoes autophosphorylation at T286 rendering the kinase partially independent of Ca^2+^ (autonomous) and strengthening NMDAR binding (reviewed in [8]). If CaMKII activation or T286 autophosphorylation are blocked by pharmacological or genetic means, LTP induction is prevented [9]–[11], and disruption of CaMKII binding to NR2B impairs LTP and learning [12]–[14]. On the other hand, CaMKII autophosphorylation at T305/306 negatively regulates Ca^2+^-dependent activity and PSD association, suggesting a complex CaMKII modulation during synaptic potentiation and learning [15].

Prior work has shown that αCaMKII enrichment is highly variable among spines and that a positive correlation exists between the amount of bound kinase and synaptic strength at individual spines [16]. A progressive increase in average αCaMKII enrichment at the PSD takes place during postnatal development [17], [18] and holoenzyme ability to bind and regulate multiple PSD proteins [19], [20] suggests that it plays both enzymatic and structural roles at the synapse [8].

Taken together, this evidence indicates that CaMKII activity and its PSD localization must be tightly regulated [15].

CaMKIIN is an endogenous protein that specifically inhibits CaMKII by binding to the kinase site of interaction with NR2B [21], [22]. Two isoforms have been identified, CaMKIINα and β, showing wide but not identical distributions in the brain [23], [24]. CaMKIINs are found in CaMKII-containing cells and were first reported to be soluble proteins of around 8.0-kDa and 70% identity [23], [24]. However, later work suggested that the α-isoform may in fact be a larger protein (∼37-kDa) that localizes to the PSD [25].

CaMKIIN mRNA is rapidly (<30 min) expressed and protein up-regulated by novelty or fear learning in an isoform- and region-specific manner [24], [26]. This experience-dependent dynamical expression resembles what occurs with immediate early genes activated in response to neural stimulation, and it has been proposed that CaMKIINα, β proteins are plasticity-related proteins [15]. Interestingly, *in vitro* studies indicate that while CaMKIINβ dissociates from αCaMKII after Ca^2+^ removal, α-isoform binding to the enzyme can persist in these conditions [23]. This suggests that although both isoforms inhibit CaMKII with the same potency and specificity, only the α-isoform should efficiently block autonomous activity, thus probably affecting different kinase functions.

Peptides based on the inhibitory domain of CaMKIINα (CN peptides) preserve the full inhibitory properties on CaMKII. Moreover, both CaMKIIN and CN peptides interfere with Ca^2+^/calmodulin-induced CaMKII binding to immobilized NR2B C terminus [22]. We have shown that transient (30 min - 2 h) applications of CN peptides made cell-permeable by fusion to different cell-penetrating sequences, persistently depress synaptic strength in hippocampal slices by a postsynaptic mechanism [27], [28]. CN-induced depression was accompanied by a sustained reduction of GFP-αCaMKII bound in spines and coimmunoprecipitation assays showed a decrease in basal CaMKII-NMDAR binding in treated slices. Synaptic depression is observed for CN concentrations that reduce this interaction but not for lower concentrations that only inhibit kinase activity [28], suggesting that CN-depression is caused by destabilization of this interaction at synapses.

Remarkably, CN application brings LTP out of saturation, as transient CN treatment after induction of saturated LTP in a synaptic pathway, allows LTP reinduction in this pathway. Moreover, CN transient treatment also enhanced LTP induction in naïve pathways. These results suggest that a fraction of synaptic strength is controlled by the CaMKII-NMDAR binding and that the amount of this complex at synapses critically regulates subsequent potentiation.

Therefore, CaMKIIN emerges as a natural candidate for the regulation of both CaMKII synaptic localization and activity. Here, we further investigate this new type of synaptic depression induced by CN peptides, demonstrating that it is different from LTD. We provide evidence in support of an activity-independent, direct effect on PSD-bound CaMKII.

## Results

To further characterize the mechanism of CN-depression we used a 27 amino acid peptide derived from CaMKIINα, made cell permeable by fusion to the antennapedia sequence ant (antCN27 or ant-CaMKIINtide, [27]).

**Figure 1:**
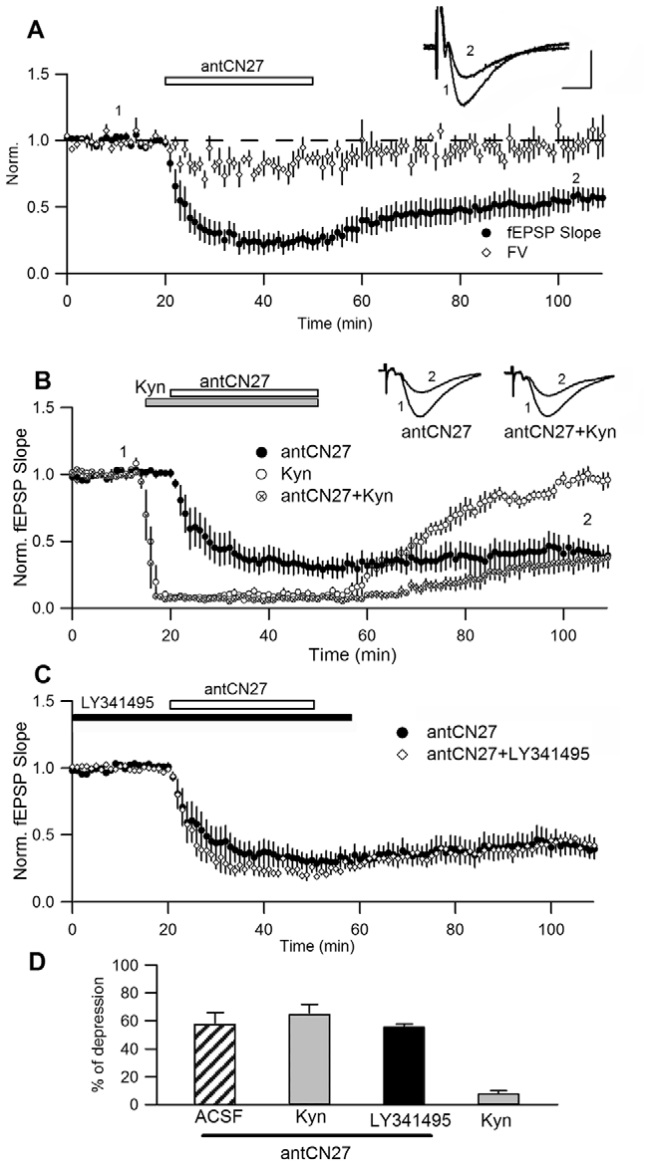
CN-induced depression is independent from synaptic activity. **A.** Persistent synaptic depression induced by transient bath application of antCN27 (5 µM, 30 min) (% depression = 45±8% = mean ± SEM, n = 5). Data were normalized relative to baseline values before drug application. A slight and reversible reduction in presynaptic fiber volley (FV) is observed only during drug application. **B.** In a series of interleaved experiments, antCN27 was applied in regular ACSF or in the presence of kynurenic acid (Kyn; 10 mM), antagonist of AMPA/kainate receptors (n = 4, each). The inhibition of transmission by Kyn itself was completely reversible (8±2%, n = 4; similar to control experiments with ACSF solution changes (see Fig. 6); t-test, p = 0.28). **C.** Preincubation with the broad mGluR antagonist LY341495 (20 mM, n = 5) had no effect on CN-depression. **D.** Summary plot of depression for the different conditions (58±8%, for ACSF; 65±7%, for Kyn and 56±5%, for LY341495; one-way ANOVA, p = 0.62). Insets in A, B are representative field potential waveforms (averages of eight consecutive recordings) obtained at the times indicated by numbers; calibration: 0.4 mV, 5 ms. Norm.: Normalized. Error bars represent SEM in all figures. % depression: average for the last 10 min of recording and relative to baseline transmission, in all figures.

In agreement with our earlier report [27], bath application of antCN27 (5 µM, 30 min) persistently reduced basal field EPSP (fEPSP) slope in rat hippocampal slices, as measured 1 h after drug washout (Fig. 1A). We already showed that this persistent effect is expressed postsynaptically, as a similar depression was observed for postsynaptic responses to locally applied AMPA [27]. Moreover, postsynaptic transfection of a closely related peptide (CN19) produced synaptic strength depression [28]. It should be noted that the larger acute effect observed during antCN27 application results from a combination of this postsynaptic depression and a mostly reversible change in presynaptic excitability, expressed as a transient decrease in fiber volley (FV) amplitude in Fig. 1A (see [27] for details). Here we will focus on the persistent postsynaptic depression of synaptic strength, measured after peptide washout.

### Synaptic Activity and Ca^2+^Signaling are Not Required for the Induction of CN-depression

Several synaptic plasticity phenomena are triggered by the activity of ionotropic and metabotropic glutamatergic receptors [29], [30]. Therefore, it becomes relevant to ask whether the activity of glutamate receptors is required for the induction of CN-depression. This was previously explored by turning off synaptic stimulation during antCN27 application [27]. When stimulation was restored after peptide removal, CN-depression was intact, suggesting an activity-independent process. However, a contribution of miniature synaptic events or of an overall increase in neural activity in the slice was not discarded by that experiment. Therefore, as a more rigorous test for a role of glutamatergic transmission in the induction or modulation of CN-depression, we applied antCN27 in the presence of antagonists of glutamatergic receptors. In a first series of experiments, antCN27 was applied 5 min after ionotropic synaptic transmission was completely inhibited by the broad spectrum and reversible glutamate receptor antagonist kynurenic acid (Kyn). We observed that in these conditions, persistent depression was apparent 1 h after removing both drugs (Fig. 1B). For comparison, the average of interleaved experiments performed in slices from the same animals but in which antCN27 was applied in regular external solution, is shown superimposed. In separate trials, we confirmed that synaptic inhibition by Kyn itself was highly reversible. Finally, in similar experiments conducted in the presence of the broad mGluR antagonist LY341495, CN-depression was also triggered (Fig. 1C). The bar plot in Fig. 1D summarizes these results, showing no statistical difference between CN-depression induced in the presence or absence of glutamatergic antagonists (see legend for details). These results show that glutamatergic synaptic activity is not required for triggering CN-depression and that it does not modulate the effect. Moreover, they also rule out the possibility that the observed depression could be due to unspecific drug effects causing an increase in slice activity and the induction of known forms of activity-dependent depression, as NMDAR- or mGluR-dependent LTD (NMDAR- and mGluR-LTD, respectively).

**Figure 2:**
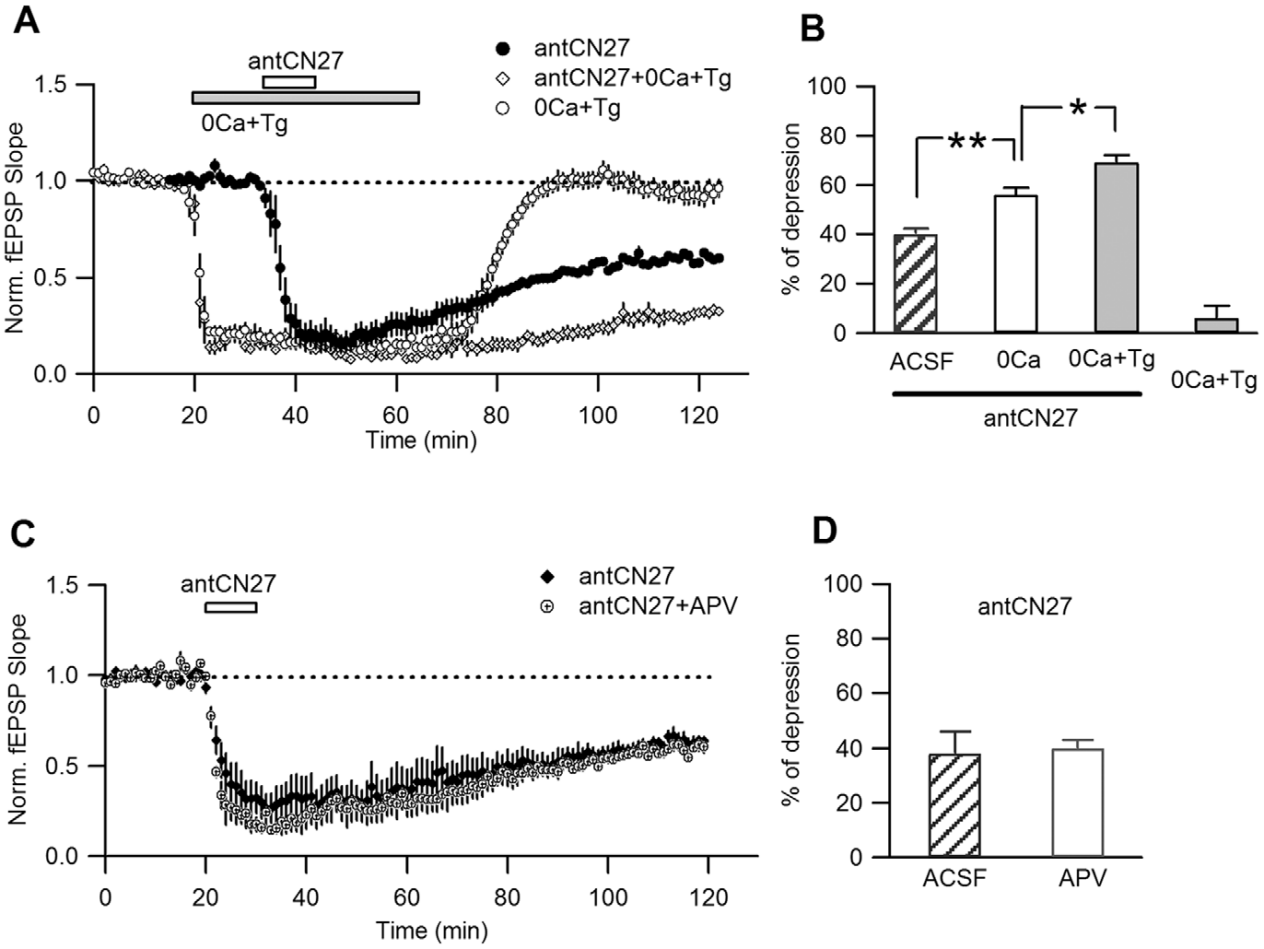
Ca^2+^ is not required for the induction of CN-depression. **A.** Depression induced by brief applications of antCN27 (5 µM, 10 min) in interleaved experiments conducted in regular ACSF (“antCN27”) or in a solution with no Ca^2+^ added and containing 10 mM EGTA and 10 µM thapsigargin, Tg (“antCN27+0Ca+Tg”). Application of the last solution by itself reversibly inhibited transmission (“0Ca+Tg”). **B.** Summary plot of percent depression induced by antCN27 in regular ACSF (40±2%, n = 4), in 0-Ca^+2^ solution without Tg (“0Ca”; 56±3%, n = 4; not shown in A) and in 0Ca+Tg solution (69±3%, n = 6). *, p = 0,02; **, p = 0.009; one-way ANOVA, post hoc Tukey HSD test. Data for application of 0Ca+Tg solution only is also shown (6±5%, n = 6. No significant difference with ACSF solution change experiments; p = 0.49, t-test). **C.** Blockade of NMDAR-dependent Ca^2+^ influx does not reproduce the effect of removing Ca^+2^, as revealed by an independent set of interleaved experiments of brief applications of antCN27 alone or in the presence of 100 µM APV during the whole experiment (n = 4, each) **D.** Percent depression for the experiments shown in C (40±3%, for APV; 38±8%, for regular ACSF, p = 0.98, t-test).

Ca^2+^ signaling is involved in several synaptic plasticity processes in CA1 as LTP, NMDAR- and mGluR-LTD [30], [31]. Although our previous results suggest that Ca^2+^ influx through NMDAR is not required for inducing CN-depression, an intracellular Ca^2+^ increase mediated by voltage-gated calcium channels (VGCCs) -which are putative targets of CaMKII modulation [32], [33]- or Ca^2+^ release from intracellular stores, could participate in CN-depression. To address these possibilities, we tested if CN-depression can be induced in the absence of extracellular Ca^2+^ and after endoplasmic-reticulum Ca^2+^ depletion. To minimize the time that the slices would be maintained in these conditions, we assessed if CN-depression can be induced by shorter antCN27 applications. As shown in Fig. 2A (filled symbols) and B, 10 min antCN27 treatment still caused significant CN-depression as measured 80 min after drug washout. In interleaved experiments, similar antCN27 applications were conducted on slices bathed by a solution with no Ca^2+^ added and containing the Ca^+2^ chelator EGTA and thapsigargin (Tg), a compound inhibiting endoplasmic reticulum SERCA-type Ca^2+^ pumps and therefore causing store depletion. Surprisingly, when antCN27 was applied in Ca^2+^-free conditions, CN-depression was significantly increased as compared to regular conditions (Figs. 2A, B). This difference was not due to unspecific effects on transmission produced by the Ca^2+^-free condition by itself, as in separate experiments we determined that synaptic inhibition by this treatment was fully reversible (Fig. 2A,B; “0Ca+Tg”). In a separate series of experiments where Ca^2+^ was removed from the extracellular solution, but Tg was not added (not shown in Fig. 2A), a significant increase in the magnitude of depression was still observed (Fig. 2B, see legend for details and statistic analysis).

The previous results indicate that Ca^2+^ is not required for the induction of CN-depression. Indeed, reducing external Ca^2+^ increases the effect and a further increment is observed when Ca^2+^ release from internal stores is prevented (see Discussion).

We showed that blockade of glutamatergic transmission did not affect CN-depression induced by 30 min antCN27 (5 µM) application (Fig. 1B), a treatment previously shown to be saturating as longer treatment with a higher concentration caused a similar affect [27]. As removing Ca^2+^ amplified depression caused by short antCN27 applications, it was possible that an activity-dependent modulation by Ca^2+^ influx through NMDARs could have been missed in saturating conditions. Therefore, we tested if the blockade of NMDAR-dependent Ca^2+^ influx could mimic the effect of external Ca^2+^ removal for short antCN27 applications. We found that in the presence of the NMDAR antagonist APV CN-depression was not affected compared to interleaved experiments conducted in regular ACSF conditions (Fig. 2C, D). Therefore, blockade of NMDAR-dependent Ca^2+^ influx does not reproduce the effect of removing extracellular Ca^+2^. This result rules out a role of NMDAR activity in the induction or the modulation of CN-depression.

### CN-depression does not Depend on Protein Synthesis or Proteasome-mediated Protein Degradation

To further investigate the mechanism of CN-depression, we evaluated if it involves complex metabolic pathways including protein synthesis or their degradation by the proteasome. Such processes have been implicated in forms of synaptic plasticity as late LTP [34], [35] and mGluR-LTD [36], [37].

**Figure 3:**
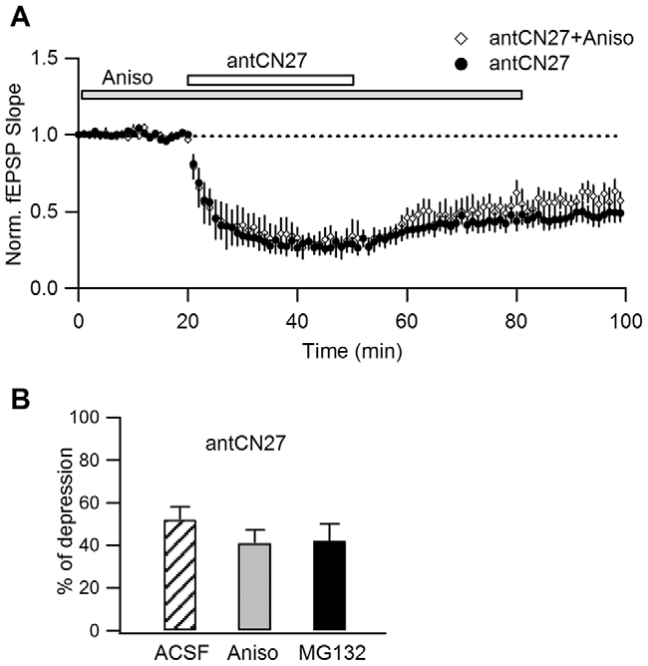
Protein synthesis and degradation are not required for CN-depression. **A.** Depression induced by antCN27 (5 µM, 30 min) in the presence of 20 µM anisomycin (Aniso) is comparable to that induced in regular ACSF. Aniso was applied at least 20 min before antCN27 and was maintained for 1 h after starting peptide treatment. **B.** Summary plot of percent depression for the experiments shown in A and for similar trials in the presence of the proteasome inhibitor MG 132 (10–20 µM) (52±6%, n = 9, for ACSF; 41±6%, n = 5, for Aniso; 42±8%, n = 4, for MG132; one-way ANOVA, p = 0.45).

To address a possible dependence on translation, we treated hippocampal slices with the cell-permeable translation inhibitor anisomycin (Aniso) that blocks protein synthesis in minutes [36], and evaluated if depression was affected by the presence of this drug. We first verified that Aniso treatment does not by itself modify basal synaptic response for at least 40 min after application (not shown). In the test experiments, Aniso was bath-applied 20 min before antCN27 and after peptide removal it was kept in the external solution until completing 1 h from the start of antCN27 treatment. Fig. 3A shows superimposed summary plots for test experiments and for a series of control experiments where similar antCN27 applications were made in regular ACSF. As observed, inhibition of protein synthesis has no effect on the time course or magnitude of CN-depression. A possible role of protein degradation mediated by the proteasome system was assessed in similar experiments conducted in the presence of the proteasome inhibitor MG132. As shown in the summary plot of Fig. 3B, CN-depression was not different in these three groups (see legend). These results indicate that CN-depression does not involve protein synthesis or proteasome-mediated degradation, at least for the time window considered.

### CN-depression and NMDAR-LTD do Not Occlude each Other

The fact that CN-depression does not require activation of NMDARs or Ca^2+^ influx indicates that the induction mechanism of this form of depression is different from NMDAR-LTD. However, to check the possibility that both forms of depression could share expression mechanisms, we conducted occlusion experiments. In these experiments, it was assessed if development of one of these forms of depression occludes or reduces the subsequent expression of the other. With this goal, NMDAR-LTD and CN-depression were sequentially induced, in this order or the other way around, and the relative depression caused by the second treatment was compared to the effect of the same treatment when applied first.

**Figure 4:**
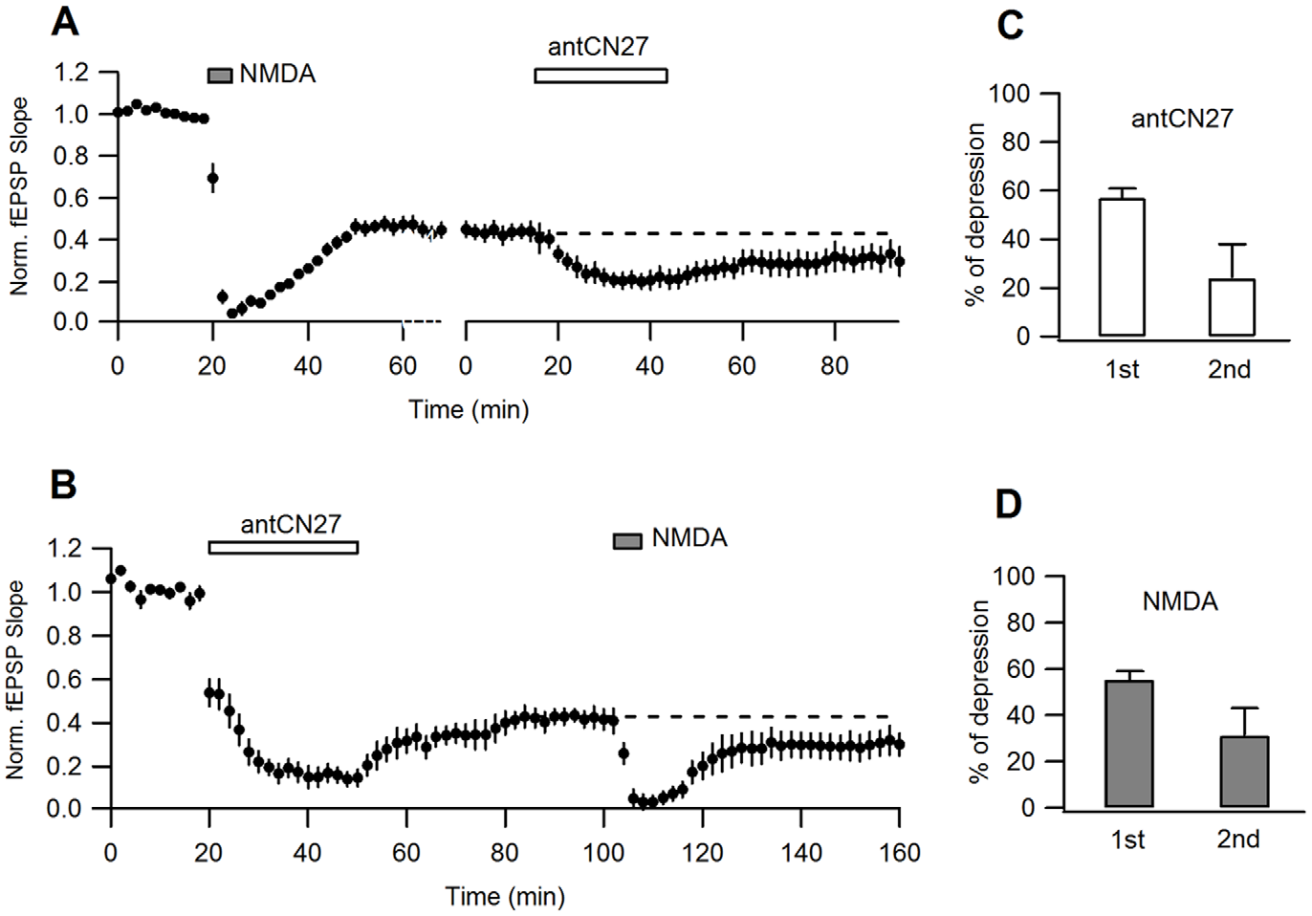
Occlusion experiments for NMDAR-LTD and CN-depression (I). **A.** LTD was induced by bath application of NMDA (20 µM, 5 min) and CN-depression was subsequently induced (5 µM, 30 min; n = 8). The data were realigned according to the time of second drug application. **B.** Similar experiments to A, with drugs applied in reverse order (n = 7). **C.** Percent depression caused by antCN27 applied after LTD (“2nd”; from A) is not significantly different to that observed in regular conditions (“1st”; from B) (57±4%, 1st; 24±14%, 2nd; measured 50 min after antCN27 removal; t-test, p = 0.06). **D.** The magnitude of LTD induced in regular conditions (“1st”; from A) and after CN-depression (“2nd”; from B), also shows no statistical difference (55±4%, 1st; 31±12%, 2nd; measured 45 min after NMDA removal; t-test, p = 0.07). To quantify depression induced by the second treatment, data were renormalized to the level of transmission before drug application.

As it was pointed out before, saturated CN-depression can be induced by 30 min applications of 5 µM antCN27 [27], therefore, we chose to use this protocol for the occlusion experiments. To induce LTD we utilized a widely-used chemical protocol, consisting of bath application of NMDA (20 µM, 5 min). As shown in Fig. 4A, this treatment caused a large depression as measured 45 min after NMDA removal (see legend). When applied after the LTD protocol, antCN27 further depressed synaptic transmission, but appropriate quantification of this depression requires renormalization of transmission to the level previous to antCN27 application, for each experiment (see below). Similarly, NMDA treatment after CN-depression further reduced transmission (Fig. 4B). Figs. 4C and D show a comparison -after renormalization of data- of the magnitude of depression caused by each treatment in naïve slices and in slices previously subjected to the other treatment. While a trend for a smaller effect for the second treatments is observed, in both cases differences were not statistically significant (see Fig. 4 legend for details).

**Figure 5:**
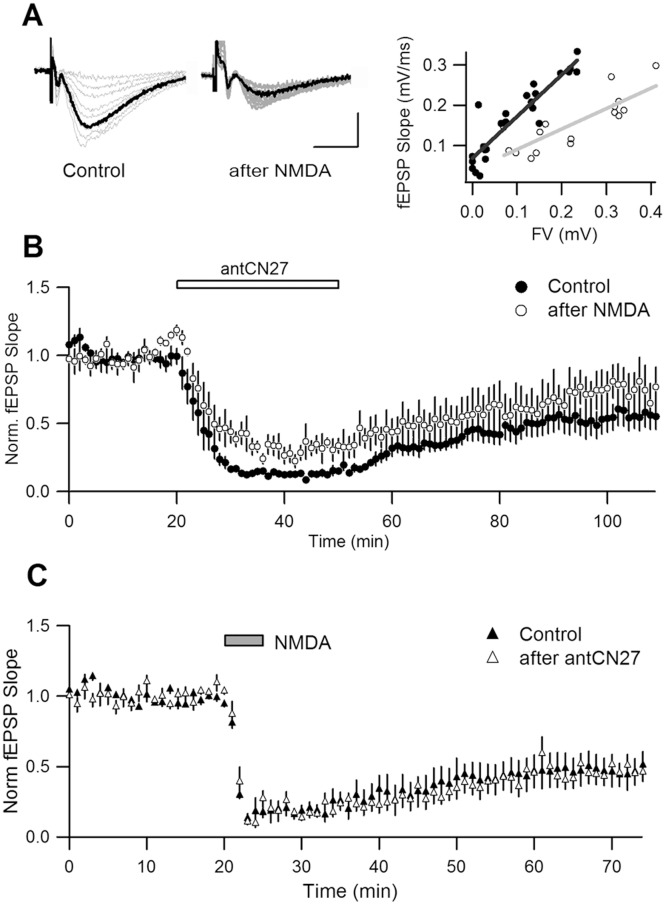
Occlusion experiments for NMDAR-LTD and CN-depression (II). Slices from the same animal were pre-incubated in interface chambers with NMDA/antCN27 (test slices) or underwent ACSF solution changes mimicking drug application and washout (control slices). Subsequently, simultaneous FP recording and application of the second treatment was conducted for pairs of test and control slices. **A, B.** Test slices were pre-incubated with NMDA (20 µM, 10 min). **A.** Left, superimposed FP traces obtained for increasing stimuli from a slice pre-incubated with NMDA and its control. Right, I-O curves. % LTD was estimated by comparing I-O slope (% LTD = 69±7%; n = 4). **B.** AntCN27 was simultaneously applied to test (after NMDA) and control slices in the recording chamber. AntCN27-induced depression (last 10 min of recordings) was comparable in both groups (25±14%, for NMDA-preincubated slices versus 44±11%, for control slices, n = 4, paired t-test, p = 0.16). **C.** Test slices were pre-incubated with antCN27 (5 µM, 30 min) and NMDA (20 µM, 5 min) was later applied to test and control slices in the recording chamber (% depression = 54±6%, after antCN27 versus 53±10%, for control slices: n = 5, t-test, p = 0.98).

This result indicates that there is no occlusion, but the high variability in the magnitude of depression induced by the treatments applied at later stages (compare error bars for early and late treatments in Fig. 4C, D) suggests that time-dependent unspecific factors could affect results when transmission was monitored for long times. In this set of experiments, we compared the effects of treatments that were applied at two different times during recording. Therefore, we designed a different experimental procedure to verify if there is occlusion or not. This time we concurrently recorded the effect of a specific treatment (antCN27 or NMDA) on two slices that were either transiently pre-incubated with the complementary drug (test group) or exposed to ACSF solution changes mimicking pre-incubation and drug washout (control group; see Methods). A double recording chamber allowed simultaneous measuring of field potentials (FPs) in two slices belonging to different groups. This design has the advantage of avoiding differences in the timing of drug application. Moreover, slices from test and control groups came from the same animal and were subjected to the same drug application during recordings, allowing pair comparison. Summary plots for these experiments are shown in Fig. 5.

In the first set of experiments we pre-incubated the slices with NMDA and applied antCN27 in the recording chamber. To confirm that pre-incubation had actually induced LTD, we compared the input/output (I/O) curves relating presynaptic FV to fEPSP slope, from test and control slices (see Fig. 5A for a representative example and Methods for details on curve construction and analysis). The slope of the I/O curves was taken as a measure of synaptic strength. On average, NMDA preincubation induced a strong depression (see legend to Fig. 5). Fig. 5B shows superimposed the effect on test and control slices of subsequent bath application of antCN27 in the recording chamber, showing that the magnitude of depression measured 1 h after drug removal was similar for both groups. The non-significant trend of NMDA-preincubated slices to display smaller CN-depression was not relevant, as when the fEPSP slope was normalized to FV amplitude, giving a more sensitive measure of synaptic strength that corrects for any possible variations in FV during the experiment, the difference between groups narrowed (% depression = 25±13%, after NMDA, versus 21±12%, control; n = 4, paired t-test, p = 0.72; not shown).

We then did a similar set of experiments but pre-incubating with antCN27 and applying later NMDA in the recording chamber. As previously reported [27], we confirmed that antCN27 pre-incubation depressed transmission, as compared to control slices (% depression = 62±7%, n = 4; not shown). We found that subsequent application of NMDA in the recording chamber produced similar depression in test and control slices, as measured 50 min after NMDA removal (Fig. 5C). In summary, the percent depression caused by CN (or NMDA) is the same whether it is the first or the second treatment.

Taken together, these results indicate that NMDAR-LTD and CN-depression have different expression mechanisms.

### The Magnitude of CN-depression Correlates with Average CAMKII Enrichment in PSD

We have shown that CN-depression is different from LTD, it does not require synaptic activity or intracellular Ca^2+^ increase and it does not involve protein synthesis or degradation. As it is accompanied by a reduction in basal CaMKII-NR2B interaction [28], it may be caused by destabilization of this interaction at synapses.

**Figure 6:**
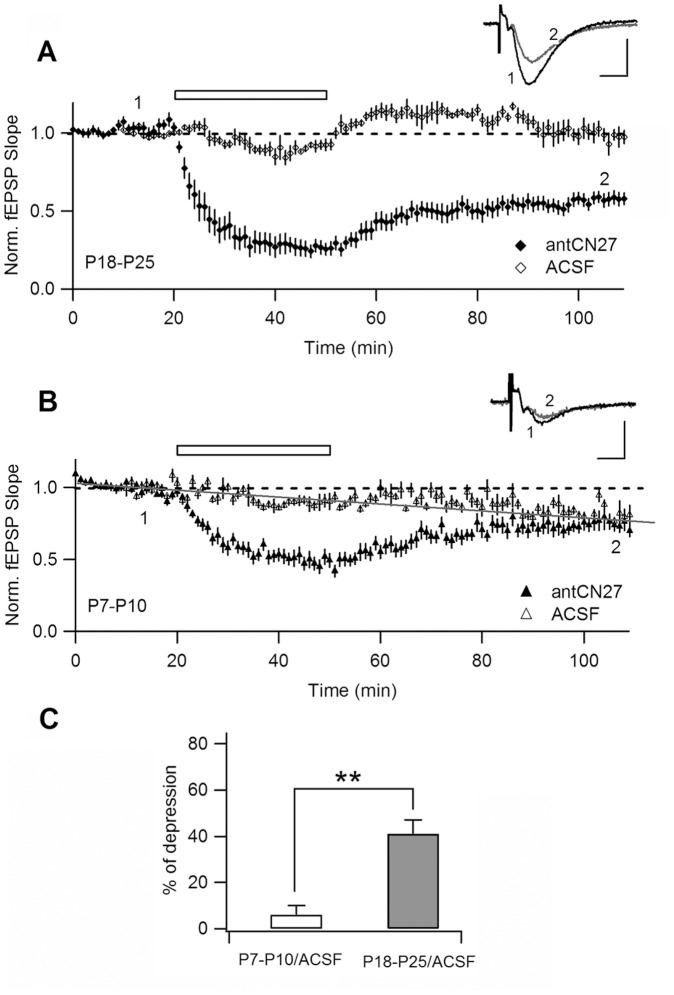
Correlation of CN-depression with average CaMKII enrichment at synapses. A. Superimposed to antCN27-induced depression in juvenile rats (42±4%, for P18–P25, n = 13) it is shown the average of control experiments conducted to evaluate signal stability during long-lasting experiments with solution recirculation. In controls (“ACSF”) no drug was applied but the solution was changed by fresh oxygenated ACSF to mimic the drug washout performed in test experiments (% rundown: 1±6%, n = 4) **B.** The same as in A, for neonate animals (P7–P10; % depression = 25±5%, n = 16). Note the rundown of synaptic potentials observed in younger rats (% rundown: 20±4, n = 7). If data is compared without correcting for rundown, depression is significantly lower in neonate rats (filled symbols in A, B; t-test, p = 0.008). **C.** Summary plot of percent decrease in transmission after antCN27 treatment (last 10 min) divided by the mean spontaneous decay measured at similar time in control experiments (**: p = 8×10^−5^, t-test). Data from 10 neonate and 11 juvenile rats.

If CN-depression is due to CN action on synaptic CaMKII, a prediction is that the magnitude of depression would positively correlate with the basal enrichment of CaMKII at PSD. Average CaMKII enrichment at synapses shows a dramatic increase during the first month of postnatal life [17], [18]. Therefore, a simple framework to test this prediction is to measure percent CN-depression in slices from animals of different ages. We hypothesized that CN-depression will be smaller in slices from young pups (P7–P10) compared to the juvenile animals (P18–P25) we used until now. Indeed, as shown in Fig. 6, antCN27 induced a significantly lower persistent depression in the younger population, as measured 1 hr after drug removal (Fig. 6A, B; filled symbols; see legend for statistics). Remarkably, the difference between ages became even more pronounced after correcting for a spontaneous signal rundown observed in long-lasting recordings in slices from young animals. As all the experiments in this paper required recirculation of a relatively small volume of external solution (see Methods) we routinely did control experiments to check stability of basal transmission. These control experiments had a similar duration as the test series, but no drug was added to the syringe and washout was mimicked by changing the external circulating ACSF by fresh solution. Fig. 6A shows superimposed test and control experiments for P18–P25 rats. After a small and stereotyped adjustment caused by solution change, basal transmission in control conditions reaches a stable level comparable to the original baseline. In contrast, in young pups control experiments revealed a rundown of synaptic transmission that has also been observed by others in neonate rats ([38]; see Discussion). In our hands, this decrease in synaptic strength becomes more evident by the end of the experiment due to the stereotyped “rebound” observed upon application of fresh ACSF solution (we drew a grey line in Fig. 6B to highlight this point). By the end of the experiments, transmission was comparable in control and test experiments, suggesting that at P7–P10 CN-depression was actually very small or absent.

Therefore, when the amount of CN-depression is calculated relative to the spontaneous signal rundown, the difference among neonates and juvenile animals turns out to be even more significant (see Fig. 6C for a summary of these results and statistical analyses). Thus, we found a direct correlation between the magnitude of CN-depression and the average amount of CaMKII in PSD, in agreement with a synaptic localization of the effect of CN peptide on CaMKII.

## Discussion

This study was directed to investigate the function of the endogenous CaMKII inhibitor proteins CaMKIIN and thereby to further understand the complex CaMKII participation in synaptic plasticity. We examined the persistent synaptic depression induced by transient treatment with CN peptides [27], [28]. We showed that CN-depression caused by antCN27 is different from known forms of activity-dependent synaptic depression. In contrast, our results point to an activity-independent, direct CN action on PSD-attached CaMKII, presumably on kinase binding to NR2B.

CaMKIIN proteins are present in regions critically involved in learning and memory, as the hippocampus and amygdala [23]. CaMKIINα mRNA is up-regulated in both places after fear conditioning [26] and CaMKIINβ transiently increases in the hippocampus after exposition to a novel context and in the amygdala after conditioning [24]. It has therefore been hypothesized that CaMKIINs may be part of a plasticity-induced negative feedback mechanism, inhibiting further CaMKII activity or its association with NMDARs [15]. Because functional studies that could shed light on the role of CaMKIIN by itself are scarce, we focused on the mechanism of CN-depression as a step necessary to unravel CaMKIIN function in hippocampal synaptic plasticity.

Our first aim was to investigate if CN-depression was activity-dependent. We knew that basal presynaptic stimulation was not required for CN-depression [27]. However, a role of spontaneous synaptic activity had not been evaluated, nor the possibility that bath application of CN peptide could transiently cause an overall increase in slice excitability and thus in synaptic activity. This could occur in the slice during general inhibition of multifunctional CaMKII activity, by altering enzyme regulation of voltage-dependent or Ca^2+^-activated channels [32], [39], [40]. Indeed, we do observe a reversible decrease in FV (Fig. 1A) suggesting a modulation of excitability in the presence of the drug. However, here we demonstrated that CN-depression was intact when the treatment was applied in the presence of antagonists of AMPARs, NMDARs or mGluRs, discarding that it could be due to NMDAR- or mGluR-dependent LTD.

Ca^2+^ plays a mandatory role in the induction cascade of several synaptic plasticity processes [30], [41]. However, we showed that common forms of Ca^2+^ signaling are not necessary for CN-depression to occur. This included Ca^2+^ influx from extracellular medium and Ca^2+^ release from endoplasmic reticulum. Intriguingly, our results revealed that lowering Ca^2+^ actually increased depression, suggesting instead that in regular conditions Ca^2+^ plays a protective role against CN-depression. This was observed even if only extracellular Ca^2+^ was removed. In these experiments we made shorter (10 min) applications of antCN27, sufficient to produce significant but not saturated CN-depression. This opened the possibility that NMDAR-mediated Ca^2+^ entry might in fact negatively regulate CN-depression, but that this effect could only be detected for non saturating treatments. Therefore, a possible activity-dependent stabilizing mechanism that opposes CN-depression was explored in non saturating conditions, with negative results: we verified that for short treatments the elimination of Ca^2+^ influx through NMDARs by receptor blockade did not mimic the “Ca^2+^ effect” and depression was intact. This thus ratified that CN-depression is not modulated by synaptic activity.

The facilitation of CN depression in Ca^2+^-free conditions may be related to the uptake mechanisms of cell-penetrating peptides (CPP). In parallel with endocytocis, CPP can directly penetrate through the plasma membrane. This transiently disturbs membranes but a repair response activated by local Ca^2+^ influx reseals them in seconds [42]. In regular conditions uptake of ant peptide by this pathway is negligible [43], but it is enhanced upon lowering extracellular Ca^2+^ [42]. It is thus possible that the increased depression observed in low Ca^2+^ conditions could be due to higher peptide uptake.

A main conclusion of these experiments is that Ca^2+^ signaling is not required for CN-depression to occur, consistent with synaptic activity-independence.

Several studies highlight the importance of protein synthesis and degradation in synaptic plasticity processes. While changes in protein metabolism are not involved in expression of NMDAR-LTD, the situation is different for mGluR-LTD [36]. This type of synaptic depression requires rapid (∼15 min) protein synthesis in the dendrites [36] and it is also regulated by proteasome-mediated protein degradation [37]. Our experiments showed, however, that CN-induced depression does not require and is not modulated by protein synthesis or proteasome-dependent degradation, at least during the explored interval of time (1 h after depression induction). Moreover, these results further indicate that CN-depression is different from mGluR-LTD.

Although we showed that CN-depression cannot be explained by the induction of NMDAR-LTD in the slices, it was still plausible that the expression mechanisms could overlap at some point. Here we used two experimental approaches to investigate if NMDAR-LTD and CN-depression occlude each other. As occlusion was not observed, we concluded that these forms of synaptic depression do not share common expression mechanisms.

In contrast, several lines of evidence indicate that the action of CN compounds could be linked to LTP phenomena, for which CaMKII activity, synaptic translocation and binding to NMDAR are critical steps (reviewed in [8]). These evidences point to the possibility that CN-depression could be caused by breakdown CaMKII-NR2B interaction at the synapse, thereby disrupting the maintenance of LTP processes that could have occurred during the life of the animal. In this scenario, CN peptides may be causing depotentiation.

Average PSD-associated CaMKII displays a dramatic increase during the first month of postnatal life [17], [18]. Therefore, a prediction was that if CN compounds actually target PSD-attached CaMKII, the magnitude of depression should be smaller for neonate rats than for juveniles. We showed that this was the case. In the absence of peptide treatment we observed the active rundown described for neonates [40], that was absent in juveniles (Fig. 6B). This phenomenon is characteristic of very young animals, it is due to active silencing of AMPA-synapses and requires basal stimulation and postsynaptic Ca^2+^ [38]. Considering this basal signal depression, we conclude that the slight decrease in FP observed in pups in CN experiments is completely explained by rundown.

Many evidences (reviewed in [44], [45]) indicate the relevance of CaMKII binding to NMDAR at PSDs for the dynamic regulation of AMPARs, at least in early stages of LTP. Our observations that CN compounds depress basal synaptic transmission and the finding that they produce an increase in individual AMPAR surface diffusion in basal conditions [46], suggest that CaMKII association to NMDAR also contributes to synaptic strength maintenance, presumably by local regulation of AMPARs properties or traffic.

Recent work shows that CaMKII activity after LTP induction is short-lasting [47], [48]. However, quantitative estimations of the different pools of CaMKII in spines suggest that such measurements would probably not reflect the small but functionally relevant NMDAR-attached fraction [49], that could preserve some Ca^2+^-independent activity. But if CN-depression relied exclusively on the ability of CN peptides to block persistent phosphorylation by autonomous CaMKII of proximal targets relevant for synaptic transmission maintenance, a recovery to basal levels would be expected after drug removal. Thus a critical condition that allows persistent depression is presumably that CN peptides are CaMKII inhibitors that also disrupt kinase binding to NR2B. CN-mediated breakdown of CaMKII-NR2B interaction would deactivate previously attached kinase subunits and may cause holoenzyme removal from synapses. Interestingly, dephosphorylation of T286 by synaptic protein phosphatase is precluded for PSD-bound CaMKII [50], possibly because binding to NR2B restricts phosphatase access [50], [51]. This may constitute a mechanism for CaMKII persistent phosphorylation at synapses that would also be disrupted by CN-induced kinase detaching from NR2B.

Finally, as the holoenzyme can simultaneously bind to multiple PSD proteins, it is conceivable that CaMKII bound to NR2B could have a structural rather than enzymatic role in the maintenance of synaptic transmission, by contributing to the existence of synaptic slots for AMPAR trapping [8].

The depotentiation hypothesis is also supported by the fact that CN allows LTP reinduction in previously saturated synaptic pathways [27], [28]. However, a result that seems at odds with this hypothesis is that in these experiments we observed that percent depression in potentiated pathways was similar to that in naïve pathways and not larger, as would be expected for an LTP reversal. This opens the intriguing possibility that CN treatment causes a cell-wide reduction in synaptic strength by a factor, independently of the previous history of Hebbian plasticity at individual synapses, thus suggesting a homeostatic effect. However, as field potentials reflect average activity of populations of synapses, additional experiments allowing resolution at the single synapse level are required to assess this possibility.

Taken together, our results are consistent with the hypothesis that a critical step in the induction of CN-depression may be a direct CN interference with CaMKII stable binding to a synaptic partner, most probably the NR2B subunit of NMDAR. This further supports a role of the CaMKII-NR2B interaction in the control of synaptic strength. The question whether this control in fact corresponds to an LTP maintenance process or to a different phenomenon, as a cell wide sliding mechanism for synaptic strength and plasticity regulation, requires more investigations. In any case, CaMKIIN action on transmission and LTP provides a mechanism to avoid saturation and keep synapses in an operative range allowing further potentiation. If the effect turns out to be preferential for previously potentiated synapses, i.e., if it depends on the previous history of Hebbian plasticity, it may constitute a mechanism for memory erasure. Future studies should also provide insight on whether CaMKIIN synthesized after training is distributed cell-wide, or at selective synapses, perhaps depending on local protein synthesis or trapping at tagged synapses. Importantly, according to our results, once the protein is available it should not require ongoing synaptic activity to produce its effect.

It has been speculated that CaMKIIN isoforms working as plasticity-related proteins [15] could contribute to CaMKII signaling termination at recently potentiated synapses. However, their effect on synaptic strength and further LTP induction suggest a more complex role and highlights important new functions of synaptic CaMKII. CaMKIIN emerges as a putative homeostatic regulator of synaptic activity and plasticity or as a molecule with the intriguing capacity to produce general or specific reversal of synaptic memory at the hippocampus.

## Materials and Methods

### Ethics Statement

Animal care and experimental procedures were approved by the Bio-Ethical Committee of the Faculty of Sciences, University of Chile, according to the ethical rules of the Biosafety Policy Manual of the National Fund for Scientific and Technological Development (FONDECYT).

### Acute Slice Preparation

Acute transversal hippocampal slices (400 µM) were prepared from neonate (P7–P10) and juvenile (P18–P25) Sprague Dawley rats, following standard dissection protocols (Moyer & Brown, 2002). For neonates, the dissection solution contained (in mM): 212.7 Sucrose, 2.6 KCl, 1.23 NaH_2_PO_4_, 25 NaHCO_3_, 10 D-glucose, 10 MgCl_2_, 1 CaCl_2_ and for juveniles: 125 NaCl, 2.5 KCl, 1.25 NaH_2_PO_4_, 25 NaHCO_3_, 10 D-glucose, 10 MgCl_2_, 1 CaCl_2_. Both solutions were ice-cooled and bubbled with 95% O_2_ and 5% CO_2_. CA3 area was removed from all the slices. Slices were allowed to recover at room temperature in a submersion chamber containing artificial cerebrospinal fluid (ACSF) composed of (in mM): 125 NaCl, 2.5 KCl, 1.25 NaH_2_PO_4_, 25 NaHCO_3_, 10 D-glucose, 1 MgCl_2_, 2 CaCl_2_, saturated with 95% O_2_ y 5% CO_2_. Slices recovered in these conditions for at least 2 h before the experiments. Between 4 and 9 slices, obtained from 3 to 6 animals, were used for each series of experiments.

### Slice Pre-incubations

In some experiments slices were pre-incubated with drugs (NMDA or antCN27; see below) in inverted interface chambers (tissue inserts, 8 µm; NUNC Brand Products), before being transferred to the recording chamber. Interface chambers were maintained in an environment saturated with 95% O_2_ and 5% CO_2_ at 30°C and slices were put on the top of the porous surface, in a drop of ACSF. For pre-incubations, this drop was carefully replaced by oxygenated solution containing the drug at the final concentration, using a pasteur pipette. To wash the drugs out, the drop solution was changed by fresh, oxygenated ACSF, four times. We followed the same procedure for control slices, but using only oxygenated ACSF. Afterwards, two slices (one per group), were transferred to the double recording chamber. As in previous experiments, slices were let to adapt during 30 min before starting recordings.

### Electrophysiological Recordings

Slices were gently transferred to a submersion recording chamber. A total volume of 10 ml of ACSF bubbled with 95% O_2_ and 5% CO_2_ in a 20 ml syringe, was re-circulated using a peristaltic pump (Masterflex). During experiments, flux and temperature were maintained at 1.5–2 ml/min and 30–31°C, respectively. Slices were allowed to adapt to these conditions during 30 min before starting recordings. Field potential (FP) recordings were obtained from CA1 stratum radiatum, by using borosilicate glass pipettes (0.3–0.6 MΩ) filled with ACSF. Schaffer collateral/comissural fibers were stimulated by bipolar concentric electrodes (FHC), placed in stratum radiatum at 250–300 µm from the recording electrode. Field potentials were recorded using a differential amplifier (A-M Systems) and 1 Hz –5 kHz band-pass filter. Stimulation was conducted using an Isostim A320 WPI stimulator. Data were acquired at 20 kHz with a PC using a National Instrument interface and Neuromatic/Igor Pro 6.03A (Wavemetrics) custom-made procedures. Stimulus duration was 0.15 ms and inter-stimulus interval was 15 s. Stimulus strength was set to produce 60–70% of the maximal response, (signal saturation or population-spike occurrence). A 20 min stable basal response was recorded before applying any treatment.

To estimate the effect of pre-incubation with drugs we compared synaptic strength in test and control slices. For NMDA preincubations, input-output (I-O) curves relating fiber volley (FV) to fEPSP slope were obtained by applying stimulation currents of increasing amplitudes (ranging from 5 to 100 µA, with steps of 5–10 µA; 3 stimulations per step). The slope of I-O curves was calculated by linear regression and values for test and control slices were compared to estimate the magnitude of LTD induced. In the case of antCN27, we checked that pre-incubations caused CN-depression (previously reported in [27]) by comparing fEPSP slope of test and control slices for the same FV magnitude.

### Data Analysis and Statistics

Igor Pro 6.03A was used to perform preliminary on-line data analysis and off-line reanalysis and statistical analyses. Synaptic strength was quantified as the maximal slope of the fEPSP rising phase. Fiber volley (FV) amplitude was measured relative to the preceding inflexion point (d^2^V/dt^2^ = 0). This method was chosen to reduce errors derived from the superposition of the stimulus artifact and the raising face of FV signals. Data were normalized by the average baseline value. In the first series of occlusion experiments (Fig. 5B), data were renormalized after recovering from the first treatment, by the 10 min average value preceding the second treatment. Igor procedures for on- and off-line analyses were developed by G. Fernández-Villalobos. In the figures, normalized data were averaged across experiments and expressed as mean (±SEM). For two-sample statistical analyses, unpaired Student’s t*-*test was used (indicated in legends as t-test), except for the occlusion experiment in Fig. 5B, where a paired t-test was used. For three or more sample tests one-way ANOVA and post hoc Tukey HSD test were applied. p<0.05 was considered as a significance criteria.

### CN Peptide and Other Drugs

Antennapedia-CN27 (antCN27, also known as Ant-CaMKIINtide and Ant-CaNtide: RQIKIWFQNRRMKWK-KRPPKLGQIGRSKRVVIEDDRIDDVLK) was obtained from the Biopolymer Synthesis Center (California Institute of Technology, Pasadena, CA). N-methyl-D-aspartic acid (NMDA) was obtained from Sigma Aldrich, and Anisomycin, EGTA, kynurenic acid, thapsigargin and MG 132 were from Tocris Bioscience.

### Drug Preparation and Application

Aliquots of the different drugs were prepared to reach the desired concentration in the circulating volume (10 ml). Anisomicin, MG-132 and thapsigargin were dissolved in DMSO (not exceeding 0.2%). antCN27 and NMDA were dissolved in bi-distilled water. Aliquots were kept at −20°C except for anisomycin which was at 2°C. For drug bath-applications, aliquots were poured in the syringe containing ACSF. The drugs were washed out by opening the recirculation circuit and passing 30 ml of fresh and oxygenated ACSF. After that, recirculation was reestablished. Kynurenic acid was dissolved immediately before experiments in a separated ACSF reservoir. The whole circulating volume was replaced by ACSF plus kynurenic acid for 35 min and then washed out with fresh ACSF as explained. For zero Ca^2+^ experiments we prepared ACSF solution with EGTA (10 mM) and containing 3 mM MgCl_2_ (to keep osmolarity constant).
